# Editorial: Actinobacteria plant interaction: recent molecular tools and biology

**DOI:** 10.3389/fmicb.2023.1209699

**Published:** 2023-05-10

**Authors:** Zhen Wang, Abhay Kumar, Pratiksha Singh, Manoj Kumar Solanki

**Affiliations:** ^1^Guangxi Key Laboratory of Agricultural Resources Chemistry and Biotechnology, Agricultural College, Yulin Normal University, Yulin, China; ^2^Department for Innovation in Biological, Agri-food and Forestry Systems, University of Tuscia, Viterbo, Italy; ^3^Guangxi Key Laboratory for Polysaccharide Materials and Modifications, Guangxi University for Nationalities, Nanning, China; ^4^Plant Cytogenetics and Molecular Biology Group, Faculty of Natural Sciences, Institute of Biology, Biotechnology and Environmental Protection, University of Silesia in Katowice, Katowice, Poland

**Keywords:** Actinobacteria plant interaction, rhizosphere microbiome, taxonomy, plant growth promotion (PGP), biological control, symbiont

Actinobacteria are Gram-positive bacteria with a high C + G DNA content and a morphology mostly intermediate between bacteria and filamentous fungi. More than 50% of the active substances, such as antibiotics, found in over 33,000 microbial secondary metabolites are derived from actinobacterial metabolites (70% of which are *Streptomyces*) (Mitra et al., 2022). Actinobacteria are important microbial resources that contain abundant naturally active substances. Despite the continuous discovery of new species, the currently isolated species account for <1% of the total number of Actinobacteria species in nature. The exploration of new Actinobacteria remains a research hotspot. Plant symbiotic Actinobacteria are widely distributed within various plant tissues as well as in the rhizosphere and leaf (Pang et al., 2022). They establish mutually beneficial relationships with plants during coevolution and do not pose any harm to the host plant. Actinobacteria promote the plant absorption of nutrients, adaptation to adversity, resistance to diseases, and maintain the balance of the plant microecology (Wang et al., 2021; Solans et al., 2022). Actinobacteria play an important role in ecological agriculture, the food and pharmaceutical industries, and environmental governance. The plant symbiotic Actinobacteria not only promote the growth and reproduction of the host plant but also produce active metabolites that are the same or similar to those of the host plant (Wang et al., 2023). Regarding fully developed microbial resources, further exploration of plant symbiotic Actinobacteria is of great significance.

This Research Topic focuses on further understanding recent research on the interaction between plants and Actinobacteria. This Research Topic includes five original articles on plant species, such as *Coriaria myrtifolia, Dioscorea opposita* Thunb. (yam), *Camellia oleifera, Aconitum carmichaelii, Gentiana rigescens, Daphne aurantia, Oxytropis falcata*, and microbial genera such as *Arthrobacter, Frankia, Streptomyces*, and *Herbiconiux*. In the context, Swanson et al. found that the neurotoxicity and wild nature of *C. myrtifolia* does not greatly shape the plant microbiome. *C. myrtifolia* flora is dominated by Cyanobacteria in the leaves, stems, and fruits, and Actinobacteria and Proteobacteria in the root and nodule organelles. Nodules are a special nitrogen-fixing ecological niche mainly inhabited by *Frankia* in this plant but containing several non-*Frankia* bacteria. In addition to *Frankia* cluster 2, the presence of clusters 1 and 4, and many cluster 3 strains was also detected in the nodules, roots, and rhizosphere of *C. myrtifolia*. The filtration effect of the rhizosphere and nodules was observed. All members of the four *Frankia* clusters appear in the roots, indicating that these microbiota have different growth-promoting potential and may be the main refuge for the proliferation of all *Frankia* clusters.

N,N-dimethylhexadecylamine (DMHDA) induces iron deficiency, plant defense responses, and quorum sensing (QS) in *Arthrobacter*. *Arthrobacter* sp. UMCV2 promotes plant growth by releasing the volatile organic compound DMHDA. Chávez-Moctezuma et al. assembled a draft genome of *Arthrobacter* sp. UMCV2 and found that it does not belong to any previously described species. Genomic exploration revealed the presence of 16 *luxR*-related genes, but no *luxI* homologs were found. Among them, 11 gene sequences have *LuxR* characteristic DNA-binding domains and a helix-turn-helix motif, and are designated as autoinducer-related regulators (AirR). These four sequences have *LuxR* like domains and are designated autoinducer-analogous regulators (AiaR). When DMHDA was used to induce cluster movement, eight *airR* and two *aiaR* genes were upregulated. When QS is triggered, DMHDA induces the expression of multiple *luxR* related genes in *Arthrobacter* sp. UMCV2.

Zhou et al. isolated 116 endophytic actinomycetes from surface-sterilized yam tissues from a yam garden in Hainan Province, China. A total of 23 isolates showed antagonistic activity against *Colletotrichum gloeosporioides*, which can cause yam anthracnose. Subsequently, the endophytic actinomycete HNM0140T, which has strong antifungal activity, multiple biological controls, and plant growth-promoting (PGP) characteristics, was identified. Treatment with strain HNM0140T significantly reduced the severity and incidence of yam anthracnose. Based on phylogenetic, genomic, and phenotypic analyses, strain HNM0140T represents a new species of *Streptomyces*, named *Streptomyces internecica* sp. nov. Genomic analysis showed that *S. internecica* sp. nov HNM0140T strain carries 18 putative biosynthetic gene clusters, some PGP-related genes, and several genes encoding antifungal enzymes.

*Camellia oleifera* is a unique edible oil crop that grows in the hilly and mountainous areas of southern China. Although *C. oleifera* is classified as a drought-resistant tree species, drought remains the main factor limiting its growth during summer and fall. He et al. found that the endophytic strain *Streptomyces albicans* OsiLf-2 alleviates the negative effects of drought stress on *C. oleifera*, thereby improving the seed, oil, and fruit quality. Microbiome analysis showed that *S. albicans* OsiLf-2 treatment significantly affected the microbial community structure of the rhizospheric soil in *C. oleifera*, reducing the diversity and abundance of soil microorganisms. Transcriptome and metabolome analyses revealed that *S. albicans* OsiLf-2 protects plant cells from drought stress by reducing water loss in root cells and synthesizing osmoregulatory substances, polysaccharides, and glycols in the roots. In addition, *S. albicans* OsiLf-2 can induce host resistance to drought stress by increasing the activity of *C. oleifera* peroxidase and antioxidant synthesis. Multiomic joint analysis of the microbiome, transcriptome, and metabolome showed that *S. albicans* OsiLf-2 contributes to the role of *C. oleifera* in resisting drought stress.

Deng et al. obtained five gram-positive, aerobic, and non-motile actinobacterial strains from different ecosystems related to four Chinese medicinal herbs (*Aconitum carmichaelii, Gentiana rigescens, Daphne aurantiaca*, and *Oxytropis falcata*) and named them CPCC 205763^T^, CPCC 203386^T^, CPCC 205716^T^, CPCC 203406^T^, and CPCC 203407, respectively. Based on the phylogenetic tree analysis of the 16S rRNA gene sequence and core genome, as well as the analysis of overall genome relatedness indices (ANI and dDDH values) and phenotypic properties (morphological, physiological, and chemotaxonomic characteristics), all five strains were determined to belong to the *Herbiconiux* genus, representing four new species. In the genomes of these five strains, putative genes coding for amidase, endoglucanase, phosphatase, and superoxide dismutase were retrieved, and these genes were classified as biosynthetic genes/gene clusters related to the PGP function. IAA production, cellulose degradation, and antioxidant experiments further confirmed their potential PGP function. Genomic analysis using *Herbiconiux* supported the results of the polyphasic taxonomy and confirmed their biological functional potential.

Currently, research on plant-related Actinobacteria is less than that on fungi and other bacteria. Due to the complex synergistic growth relationship between host plants and Actinobacteria, many issues still need to be addressed when studying their interactions. Future studies need to combine pure culture with next-generation sequencing technology, combining bioinformatics with molecular biology and organic chemistry to obtain more strains and clarify specific metabolic pathways and ecological roles. As an important microbial resource, plant-related Actinobacteria have broad application prospects, huge development space, and profound scientific research significance in agriculture, medicine, and industry. Studies on their development and utilization still have a long way to go.

## Author contributions

All authors listed have made a substantial, direct, and intellectual contribution to the work and approved it for publication.
